# Glochidial infection by the endangered *Margaritifera margaritifera* (Mollusca) increased survival of salmonid host (Pisces) during experimental *Flavobacterium* disease outbreak

**DOI:** 10.1007/s00436-021-07285-7

**Published:** 2021-08-24

**Authors:** M. Motiur R. Chowdhury, Amitav Roy, Kalle Auvinen, Katja Pulkkinen, Hanna Suonia, Jouni Taskinen

**Affiliations:** 1 Department of Biological and Environmental Science, University of Jyväskylä, P.O. Box 35, 40014 Jyväskylä, Finland; 2 Department of Mathematics and Statistics, University of Jyväskylä, P.O. Box 35, 40014 Jyväskylä, Finland

**Keywords:** Brown trout, Co-infection, Pathogen, Resistance, Unionida, Virulence

## Abstract

Co-infections are common in host-parasite interactions, but studies about their impact on the virulence of parasites/diseases are still scarce. The present study compared mortality induced by a fatal bacterial pathogen, *Flavobacterium columnare* between brown trout infected with glochidia from the endangered freshwater pearl mussel, *Margaritifera margaritifera*, and uninfected control fish during the parasitic period and after the parasitic period (i.e. glochidia detached) in a laboratory experiment. We hypothesised that glochidial infection would increase host susceptibility to and/or pathogenicity of the bacterial infection. We found that the highly virulent strain of *F. columnare* caused an intense disease outbreak, with mortality reaching 100% within 29 h. Opposite to the study hypothesis, both fresh ongoing and past infection (14 months post-infection) with glochidia prolonged the fish host’s survival statistically significantly by 1 h compared to the control fish (two-way ANOVA: fresh-infection, *F*_1, 82_ = 7.144, *p* = 0.009 and post-infection, *F*_1, 51_ = 4.227, *p* = 0.044). Furthermore, fish survival time increased with glochidia abundance (MLR: post-infection, *t* = 2.103, *p* = 0.045). The mechanism could be connected to an enhanced non-specific immunity or changed gill structure of the fish, as *F. columnare* enters the fish body mainly via the gills, which is also the glochidia’s attachment site. The results increase current knowledge about the interactions between freshwater mussels and their (commercially important) fish hosts and fish pathogens and also emphasise the importance of (unknown) ecosystem services (e.g., protection against pathogens) potentially associated with imperilled freshwater mussels.

## Introduction

Free-living individuals are likely to be infected by several parasitic species and pathogens, although most studies on the subject focus on a one host–one parasite interaction (Rigaud et al. 2010). A parasitic infection can either directly or indirectly influence any subsequent infections (Cox 2001; Johnson et al. 2015; Vaumourin et al. 2015; Kotob et al. 2016; Gopko et al. 2018). This influence can be positive, negative or neutral (Vaumourin et al. 2015; Chowdhury et al. 2017; Klemme and Karvonen 2017). In fishes, parasitic infections increase the host’s risk of secondary infections and can act as a vehicle for the transmission of bacteria to fish (Kotob et al. 2016). For instance, the monogenean parasite *Dactylogyrus intermedius* increases the susceptibility of goldfish, or *Carassius auratus*, to *Flavobacterium columnare*, resulting in higher mortality when compared to non-parasitised fish (Zhang et al. 2015). Similarly, rainbow trout co-infected with *Diplostomum pseudospathaceum* experienced higher host mortality when exposed to bacteria compared with single infections (Louhi et al. 2015).

*Flavobacterium columnare* causes columnaris disease (warm water disease) in fish, including salmonids. This pathogen can cause remarkable economic losses in fish farming due to the high mortality associated with the disease (Wagner et al. 2002; Pulkkinen et al. 2010). *Flavobacterium columnare* is an opportunistic fish pathogen that can also grow outside of the fish host (Kunttu et al. 2012). *Flavobacterium columnare* strains differ in their virulence (Suomalainen et al. 2006; Pulkkinen et al. 2018) and are capable of causing up to 100% mortality in juvenile salmonids (Suomalainen et al. 2005). Since there is no effective vaccination available for young salmonids (Sundell et al. 2014), the only treatment against *F. columnare* is antibiotics (Rach et al. 2008).

Freshwater mussels, including the freshwater pearl mussel *Margaritifera margaritifera*, are critically endangered in Europe IUCN (2019). They have declined worldwide due to habitat destruction, loss of fish hosts, siltation, pollution, invasive species and over-exploitation (Bauer 1986; Lopes-Lima et al. 2017). The life cycle of *M. margaritifera* in Europe includes an obligatory host-specific parasitic period in the gills of the Atlantic salmon *Salmo salar* or the brown trout *S. trutta* (Salonen et al. 2016, 2017) that lasts for 9–11 months (Salonen and Taskinen 2017). Therefore, the success of restoration of the endangered *M. margaritifera* is entirely dependent on the success of glochidium larvae in salmonid fish host. When matured and metamorphosed, the glochidia detach from the fish host and drop to the bottom of the river as juvenile mussels, where they begin their benthic life, which lasts up to 200 years (Helama and Valovirta 2008). Freshwater mussels are involved in many ecosystem functions and services such as biofiltration, nutrient cycling and storage, food web dynamics and bottom quality modification, leading to improved water quality, habitat structure and biodiversity, in addition of direct provision of food, tools and jewellery (Vaughn & Hakenkamp 2001; Strayer 2014; Vaughn et al. 2008; Haag 2012; Vaughn 2018). Furthermore, filtration by unionid mussel (*Anodonta anatina*) reduced the density of the cercarial larvae of the harmful fish parasite *Diplostomum* (Gopko et al. 2017b). Therefore, the decline of *M. margaritifera* can potentially induce changes in ecological interactions and services in aquatic ecosystems.

Infection by *M. margaritifera* glochidia has many adverse effects on the fish host such as hyperplasia and gill filament fusion, reduced swimming capability, increased mortality, altered thermoregulation, reduced foraging, decreased activity, lowered growth rate and social dominance and increased metabolic rate (Treasurer and Turnbull 2000; Taeubert and Geist 2013; Österling et al. 2014; Thomas et al. 2014; Filipsson et al. 2016, 2017, Chowdhury et al. 2019; Horký et al. 2019. Furthermore, brown trout infected with *M. margaritifera* glochidia had an increased susceptibility to subsequent infection with the trematode parasite *D. pseudospathaceum* (Gopko et al. 2018). Hence, it can be expected that exposing fish to *F. columnare* — and the pathogenicity and virulence of *F. columnare* during the disease outbreak — would be higher in glochidia-infected individuals than in uninfected fish, as gills are the leading and first site of infection by *F. columnare* (Declercq et al. 2013, 2015). In contrast, Ziuganov (2005) proposed that *M. margaritifera* infection could stimulate healing of the fish host *Salmo salar*, e.g. from hook wounds, and provide resistance against epitheliomata and cutaneous mycoses; however, the researcher’s empirical evidence to support this idea was limited.

The co-infection interactions between brown trout, *M. margaritifera*, and bacterial disease have not been studied. Therefore, the present study investigated the impact of *M. margaritifera* infection on the susceptibility of fish to — and on the pathogenicity/virulence of — *F. columnare* infection. The hypotheses were that (i) a previous *M. margaritifera* glochidia infection would decrease brown trout survival when exposed to *F. columnare*, (ii) decreased survival would be dose-dependent (depending on the number of glochidia) and (iii) decreased survival would be the highest among the fish from which the metamorphosed glochidia have dropped off, as glochidia detachment involves rupturing the gill epithelium, presumably helping the bacterium to enter the gill tissues.

## Materials and methods

In order to challenge the two groups (the glochidia attached and glochidia detached) of brown trout with *F. columnare* at the same time, the first experiment to study the post-infection period (glochidia detached) was started by infecting brown trout with *M. margaritifera* glochidia 1 year before the second experiment to study the period of fresh infection (glochidia attached).

### Post-infection experiment

To study the effect of *M. margaritifera* infection on the susceptibility of fish to *F. columnare* — and on the pathogenicity/virulence of bacterial infection — after the glochidia had detached (the post-parasitic period), a total of 300 (0 + years old) brown trout (Rautalampi strain) were transported from the Laukaa Fish Farm (Natural Resources Institute Finland) to the Konnevesi Research Station, University of Jyväskylä, on 25 August 2016. Laukaa and Konnevesi are located in a watershed that is not inhabited by *M. margaritifera*, but five fish individuals were dissected and verified microscopically to ensure that they did not have glochidia. The rest of the fish were allocated randomly into two 163-L flow-through tanks. Two weeks later, fish in one of the tanks were exposed to 5.0 × 10^5^ M*. margaritifera* glochidia (mass exposure, approximately 3300 glochidia per fish) that were collected on the same day from the River Haukioja in northern Finland. The control group in the other tank was exposed to an equal volume (1.5 L) of filtered glochidia suspension without glochidia (see Chowdhury et al. 2017). Before the 1.5-h exposure at 14.3 °C, the water volume in the tanks was reduced to 70 L, the water flow was stopped and aeration was provided. The success of glochidia infection was checked by dissecting three fish individuals 3 days after exposure. All the primed fish were infected with glochidia, the average ± SE abundance of infection being 1421 ± 210 M*. margaritifera* glochidia per fish.

In July 2017, 9 months after infection, the fish were individually measured (length and weight), marked with passive integrated transponder (PIT) tags (7 × 1.35 mm, Loligo Systems, Denmark) and examined for *M. margaritifera* glochidia with the naked eye (see, Salonen and Taskinen 2017); they were anesthetised using MS-222. After marking, mixed groups of infected and control fish were established so that 123 fish (the rest of the fish were used for another experiment) were allocated randomly into two replicate tanks (34 infected + 28 control fish; 34 infected + 27 control fish) to be maintained until they were exposed to *F. columnare* in November 2017. Four fish from the glochidia-infected group and two fish from the control group died before the challenge with *F. columnare*.

### Fresh-infection experiment

To study the effect of *M. margaritifera* infection on fish’s susceptibility to *F. columnare* — and on the pathogenicity/virulence of bacterial infection — during the parasitic period, when the glochidia are attached to the fish’s gills, a group of fish (200 brown trout, 0 + year old, Rautalampi strain from the Laukaa Fish Farm, Natural Resources Institute Finland) was transported to the Konnevesi Research Station in late August in 2017. The fish were randomly allocated into two 163-L flow-through and were verified as being uninfected by glochidia by dissecting the gills of five individuals. The fish in one of the tanks were exposed to *M. margaritifera* glochidia on 2 September 2017, at 14.4 °C, as described above, but with a suspension of 4.0 × 10^5^ glochidia (mass exposure, approximately 4000 glochidia per fish) collected on the same day from River Jukuanoja in northern Finland. The control group in the other tank was exposed to an equal volume of filtered glochidia suspension without glochidia. Three brown trout from the *Margaritifera*-infection tank were examined for glochidia 3 days after exposure. All the primed fish were infected, the average ± SE abundance of infection being 1,041 ± 43 glochidia per fish. Later, on 19 September 2017, all the fish were marked via fin clipping while anesthetised using MS-222. They were then reallocated randomly, similar to the post-infection experiment, into two replicate (163 L) flow-through tanks containing both infected and control fish (47 infected + 43 control fish; 47 infected + 44 control fish). They were maintained under these conditions until the challenge with *F. columnare* in November 2017. One fish from each group died before the bacterial challenge.

### Challenge with *F. columnare*, survival monitoring and bacterial infection detection

Even though the experiments focusing on the period when the glochidia were attached to the gills (fresh-infection experiment, see above) and on the period when the glochidia had detached (post-infection experiment, above) were separate experiments, their start was timed so that challenging the brown trout with *F. columnare* could be performed simultaneously. The *F. columnare* strain B549 used in the experiment was isolated from Lake Kuuhankavesi, Central Finland, in 2013 and stored at − 80 °C in a solution containing 10% foetal calf serum and 10% glycerol. The strain was revived by culturing it in a modified Shieh medium (Song et al. 1988) at room temperature under agitation (120 rpm) overnight. The revived culture was further sub-cultured in the same conditions three times into a larger medium volume in a ratio of 1-part bacterial culture to 10 parts fresh medium to obtain a sufficient concentration for the experimental exposures. The strain had been tested and found to be highly virulent in previous rainbow trout challenges (Aaltonen et al*.* unpublished).

During the week before the challenge, the water temperature in the fish tanks (fresh- and post-infection experiments) was slowly increased from 4.5 to 18 °C, which was the challenge temperature, as infection with *F. columnare* (warm water disease) is not effective in cold water (Pulkkinen et al. 2010). In both the post-infection and the fresh-infection experiments, the challenge with *F. columnare* began on 16 November 2017. *Flavobacterium* challenge was performed in an isolated tank room to avoid contamination of the facility. The fish were allocated randomly into four replicate bacterium-challenge tanks and four replicate unchallenged control tanks in both experiments, for a total of 16 × 80 L tanks with a water volume of 50 L in each. In the post-infection experiment, there were 8 *Margaritifera*-infected and 5–7 control fish mixed per tank. In the fresh-infection experiment, there were 11–13 *Margaritifera*-infected and 10–11 control fish mixed per tank. The combined number of fish per tank varied between 13 and15 (post-infection experiment) and 21 and 23 (fresh-infection experiment); the total number of bacterium-challenged/control fish was 60/57 (post-infection experiment) and 90/89 (fresh-infection experiment). Within the bacterium-challenged fish, the total number of glochidia-infected/uninfected control fish was 32/28 (post-infection experiment) and 47/43 (fresh-infection experiment). Among the unchallenged fish, the total number of glochidia-infected/uninfected control fish was 32/25 (post-infection experiment) and 46/43 (fresh-infection experiment). Rich aeration was provided but water was not changed in the 80-L challenge tanks during the experiment.

Challenge infection was started by adding 500 mL of the bacterial culture to each of the challenge tanks so that the final bacterial cell concentration was 1.0 × 10^4^ CFU mL^−1^ (continuous challenge method, Kinnula et al. 2015). An equal volume of sterile modified Shieh medium was added to the control tanks.

The fish were initially monitored for signs of bacterial infection and morbidity at 1-h intervals, but after the first morbid fish was detected, monitoring was continuous. Upon detecting signs of morbidity (mainly, when the fish swam dorsal side down continuously), the fish were removed, anesthetised with MS-222 and killed with a blow to the head. Bacterial samples were taken from the gills of each dead fish with a sterile loop and cultured on agar plates with modified Shieh medium and tobramycin to selectively isolate *F. columnare* (Decostere et al. 1997). The samples were then incubated for 48 h at room temperature. The plates were checked for the growth of yellow rhizoid colonies characteristic of *F. columnare.* The experiment ended after 29 h when all the fish from the challenge tanks had been removed as dead/moribund. After this, the remaining (unchallenged) fish were killed with an overdose of MS-222. To avoid unwanted contamination of the research station facilities with the virulent *F. columnare*–diseased fish, all the fish were immediately disposed of without taking further measurements (fish size) or examining the gills for glochidia. However, at the time of bacterial challenge, fish in the post-infection experiment were 1 + year of age and their mean ± SE length was in July 2017 (4 months before bacterial challenge) 109.64 ± 0.85 mm, while fish in the fresh-infection experiment were 0 + year of age and their mean ± SE length was 69.11 ± 0.48 mm in September 2017 (2 months before the bacterial challenge).

### Statistical analyses

Practically, all fish mortality was associated with the *F. columnare* challenge, as there was only one fish from the unchallenged *Flavobacterium*-control tanks that died. Therefore, only the *Flavobacterium*-challenged tanks were included in the statistical analyses to investigate the dependence of brown trout mortality on *M. margaritifera* infection.

### Post-infection experiment

In the post-infection experiment, the effects of glochidia infection, possible tank effect and the possible effect of fish size (weight) on survival time of the fish (response variable) were analysed using two-way analysis of covariance (ANCOVA). The model assumptions of two-way ANCOVA — independence of observations, normal distribution of the dependent variable in all subpopulations, covariate linearity, regression slope homogeneity and that all subpopulations had the same variance (homoscedasticity) — were checked before the analysis. The assumption about the homoscedasticity of the subpopulations was checked using Levene’s test (Levene = 0.917, df1 = 7, df2 = 52, *p* = 0.501). The assumption of normality was examined graphically and by using the Shapiro–Wilk test, as the number of individuals in each subpopulation was between 10–13; it was met in all the subpopulations (Shapiro–Wilk, *p* ≥ 0.118) except for one (Shapiro–Wilk, *p* = 0.030). Due to the robust nature of two-way analysis of variance (ANOVA), this slight deviation from the normal distribution in one subpopulation is not problematic regarding the accuracy of the results; however, it should be considered when interpreting the results (Leik 1997). The assumptions of covariate linearity and regression slope homogeneity were checked graphically and by performing two-way ANCOVA; the model included all of the possible two-way interactions and a three-way interaction (infection*tank, infection*weight, tank*weight, infection*tank*weight) instead of using a full factorial load. The results suggested that both assumptions were also met (all *p*-values ≥ 0.328).

Multiple linear regression analysis was performed to determine how the number of glochidia (i.e. the intensity of glochidial infection) and fish size (weight) might affect fish survival times based on fish size and number of glochidia measured during the PIT tagging. The model assumptions (normality, homoscedasticity and linearity of the residuals) were checked graphically by examining the residual plots; the independence of the residuals was checked using Durbin–Watson statistics (*d* = 2.130). All the model assumptions were met, and there was no multicollinearity problem in this model.

### Fresh-infection experiment

In the fresh-infection experiment, the effects of glochidia infection and the possible tank effect on the survival time of the fish (response variable) were analysed using two-way ANOVA. Before conducting the analysis, all the model assumptions were checked as above. According to the Shapiro–Wilk test (*W* = 0.736, *df* = 11, *p* = 0.001), one of the subpopulations did not appear to be normally distributed; this also held when examined graphically. The rest of the subpopulations were normally distributed both graphically and via the Shapiro–Wilk test (all *p*-values ≥ 0.185). As before, this slight deviation from a normal distribution does not jeopardise the reliability of the results (Leik 1997). The assumption of homoscedasticity of subpopulations was met (Levene = 0.598, *df1* = 7, *df2* = 82, *p* = 0.756).

## Results

Challenge with the bacterium resulted in a strong disease outbreak — when assessed over both experiments, the first morbidity was observed after 17 h and mortality among the *F. columnare*–challenged tanks reached 100% within 29 h. However, none of the fish that were not challenged with *F. columnare* died (four tanks in the post-infection experiment and four tanks in the fresh-infection experiment), except for one individual. While *F. columnare* could be isolated from all individuals from the *F. columnare*–challenged tanks, the bacterium was not isolated from the single unchallenged fish that died.

### Post-infection experiment

**Fig. 1:**
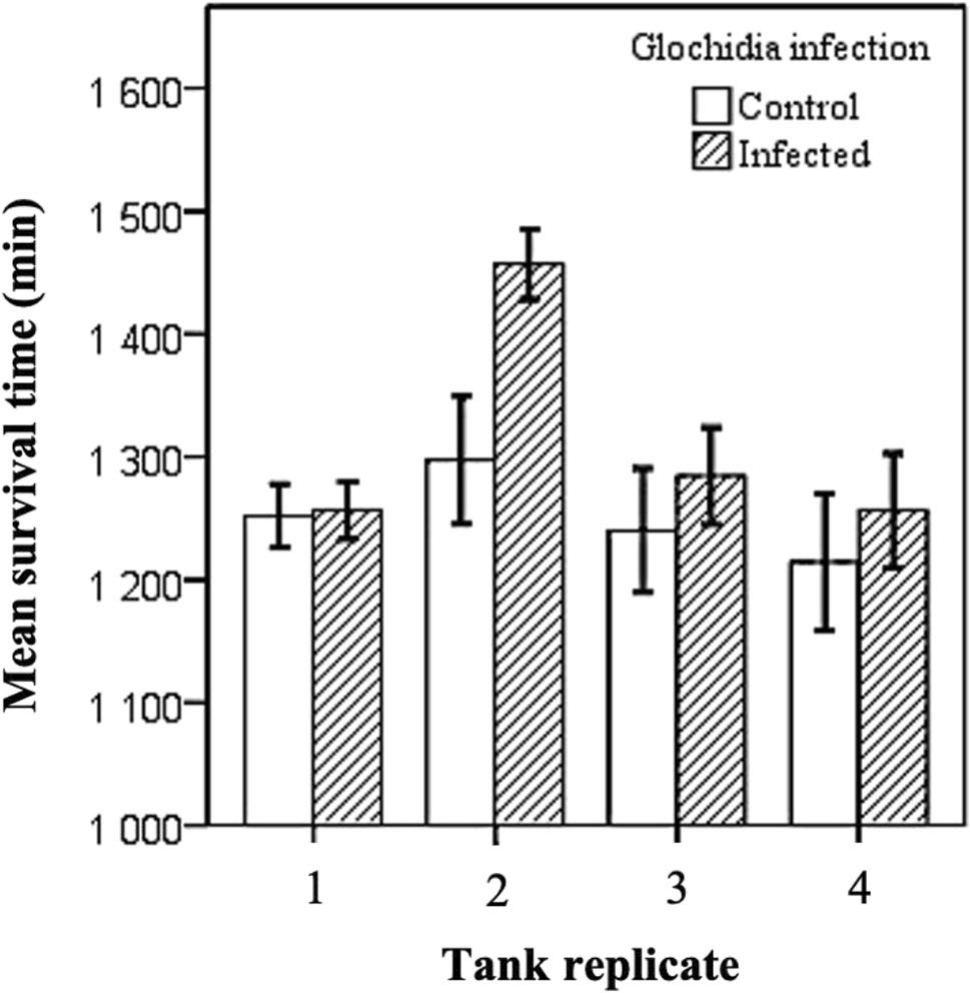
Tank-specific mean ± SE survival times of brown trout previously infected with *Margaritifera margaritifera* glochidia and those of uninfected control brown trout in the ‘post-infection experiment’, where fish were challenged with *Flavobacterium columnare* 14 months after exposure to *M. margaritifera* (i.e. when the glochidia had already detached from the infected fish)

The effect of the glochidia infection on fish survival time was statistically significant (*F*_1, 51_ = 4.227, *p* = 0.044). The survival time of the *Margaritifera*-infected individuals was longer than that of the control fish by an average 1 h (Fig. 1). The covariate, fish weight (measured in July, 4 months before the bacterial challenge), was not statistically significant (*F*_1, 51_ = 1.282, *p* = 0.263), suggesting that the survival time of the fish was independent of fish size (weight). In contrast, there was a statistically significant tank effect (*F*_3, 51_ = 5.156, *p* = 0.003). Tukey’s test in equivalent two-way ANOVA without the covariate fish weight indicated that one tank had a higher survival rate that differed from all the other tanks (*p* < 0.029 in all comparisons between tanks) (Tank 2, Fig. 1). However, the interaction between glochidia infection and tank was not statistically significant (*F*_3, 51_ = 1.330, *p* = 0.275), indicating that the effect of glochidia infection was parallel to increased survival time in all tanks.

**Fig. 2:**
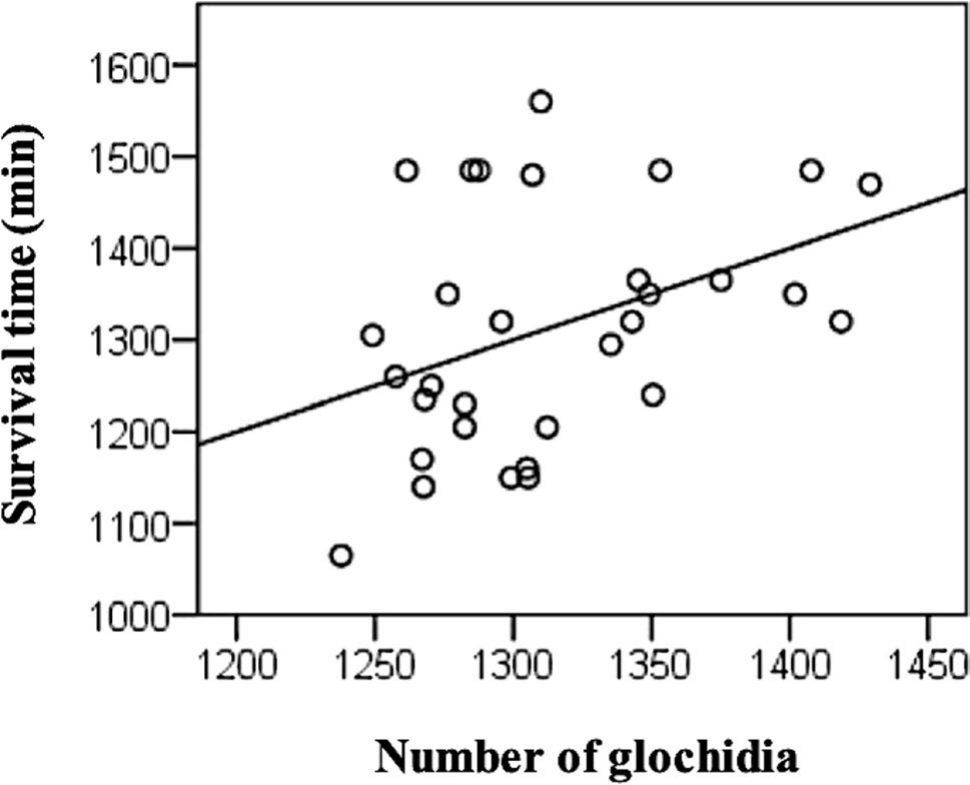
Survival time of brown trout previously infected with *Margaritifera margaritifera* glochidia as plotted against the unstandardised predicted value of the number of glochidia in brown trout, according to results of the multiple linear regression analysis (line), in the ‘post-infection experiment’ where fish were challenged with *Flavobacterium columnare* 14 months after exposure to *M. margaritifera*. In this experiment, the numbers of glochidia were counted 4 months before the bacterial challenge (before the glochidia detached)

Multiple linear regression indicated a statistically significant positive association between the number of glochidia (counted in July before challenge exposure) and survival time of *Flavobacterium*-challenged fish (*t* = 2.103, *p* = 0.045). However, multiple linear regression did not indicate any association between glochidia number and fish weight (*t* = 1.677, *p* = 0.105). The resulting regression model was.

$$\mathrm{Survivaltime}=1094.382+0.097*\mathrm{Glochidiaintensity}+12.173*{\mathrm{Fishweight}}_{\mathrm{g}}$$

with *R*^2^ = 0.159. Thus, the higher the abundance of glochidia, the longer the survival time (Fig. 2).

### Fresh-infection experiment

**Fig. 3:**
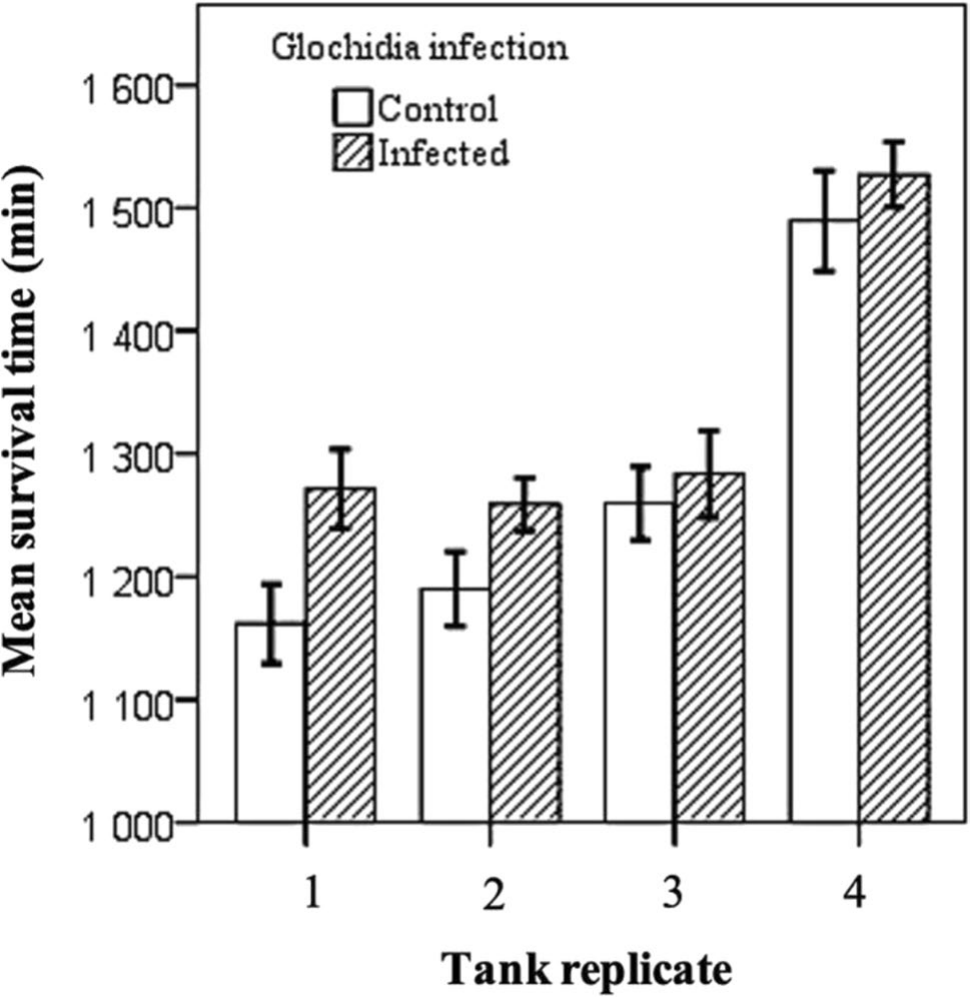
Tank-specific mean ± SE survival times of brown trout previously infected with *Margaritifera margaritifera* glochidia and those of uninfected control brown trout in the ‘fresh-infection experiment’ where fish were challenged with *Flavobacterium columnare* 2 months after exposure to *M. margaritifera* (i.e. when the glochidia had recently attached to, and not yet detached from, the infected fish)

Two-way ANOVA on the survival time of fish — with glochidia infection (infected, uninfected) and tank (four tanks; *Flavobacterium*-challenged tanks, only) as fixed factors — indicated that the effect of glochidia infection on fish survival time was statistically significant (*F*_1, 82_ = 7.144, *p* = 0.009). The survival time of the *Margaritifera*-infected fish was longer than that of the control fish (Fig. 3). There was also a statistically significant tank effect (*F*_3, 82_ = 38.557, *p* < 0.001). Tukey’s test indicated that one tank had a higher mean survival time that differed from all the other tanks (*p* < 0.001 in all comparisons between tanks) (tank 4, Fig. 3). However, the interaction between glochidia infection and tank was not statistically significant (*F*_3, 82_ = 0.722, *p* = 0.541), indicating that the effect of glochidia infection was parallel to increased survival time in all tanks.

## Discussion

Opposite to the study hypothesis, the survival time of the fish infected with *M. margaritifera* glochidia was longer than the survival time of the uninfected control fish during the *F. columnare* disease outbreak, both in the fresh-infection and in the post-infection experiments. In the post-infection experiment, the survival time of the fish increased with the number of *M. margaritifera* glochidia on the fish before the challenge with *F. columnare*. After the experiment, the bacterium *F. columnare* was isolated in every (dead) fish from the *F. columnare*–exposed tanks. Only one fish from one of the control tanks died, but *F. columnare* could not be isolated from that individual. Thus, the mortality of fish in this experiment can be specifically connected to *F. columnare*. This finding justifies two conclusions. First, the longer survival of *M. margaritifera*–infected fish, and the positive relationship between glochidia number and survival rate, indicates that the glochidia infection was associated with decreased pathogenicity (virulence) of *F. columnare* in the brown trout. Second, in these experiments, susceptibility differences between fish infected with *M. margaritifera* glochidia and uninfected control fish were not observed, as all the *F. columnare*–exposed trout acquired *F. columnare* infection regardless of glochidia infection.

All the fish challenged with this virulent *F. columnare* strain died within 29 h, and the difference in the mean survival time between *M. margaritifera*–infected and control fish was about 1 h in both experiments. The current challenge method is widely used when studying the infectivity and virulence of bacterial pathogens, including *F. columnare*, in fish; it is not exceptional that a virulent *F. columnare* strain with a relatively high bacterial dose can cause 100% mortality in juvenile salmonids within hours in experimental conditions (Kunttu et al. 2009, 2012; Kinnula et al. 2017; Pulkkinen et al. 2018). Although the survival of glochidia-infected fish was only 1 h longer than that of the control fish (on average) in this experiment with a highly virulent bacterial strain, the findings can provide a substantial survival benefit with a less virulent pathogen or with lower bacterial doses and in less stressful (natural) conditions. Thus, it is possible that *M. margaritifera* infection may decrease the pathogenicity/virulence of *F. columnare* in both natural and aquaculture conditions.

The mechanism behind the longer survival of glochidia-infected fish after exposure to *F. columnare* is not known. It could be the enhancement of an unspecific immunity within fish due to *M. margaritifera* infection. In teleost fishes, unspecific immune defence (primary immune system, innate immunity) includes cellular components (i.e. phagocytotic cells (macrophages and granulocytes)), natural killer cells and humoral components (i.e. defence molecules such as cytokines, interferons and the complement system) (e.g. Jørgensen 2014). *Margaritifera margaritifera* infection has been shown to induce transitory spleen enlargement (Thomas et al. 2014). The spleen is the major antibody-producing organ in teleost fish (Manning 1994), but spleen enlargement can also be a signal of infection — rather than a signal of enhanced immunocompetence — in fish (Seppänen et al. 2009). Furthermore, relative spleen size can decrease due to stress in fish (Kortet et al. 2003). Kunttu et al. (2009) failed to create protection against *F. columnare* in rainbow trout, even though the applied immunostimulant treatments raised the values of several parameters of innate immunity in the fish. However, immunostimulation as an explanation for the current results cannot be rejected. If the enhanced unspecific immune defence is behind the present results, it suggests that the immunostimulating effect of *M. margaritifera* glochidia is long-lasting, as the exposure to *Flavobacterium* in the post-infection (post-parasitic period) experiment took place 14 months after infection with glochidia and 3–4 months after the glochidia detached from the fish host.

*Flavobacterium columnare* enters the fish body mainly through the gills (Declercq et al. 2013, 2015). Therefore, an alternative mechanism for the protective effect of glochidia could be that the structure of the gills may change due to *M. margaritifera* infection so that the entry of the bacterium through the gills, or the establishment of the bacterium on the gills, is weakened. *Margaritifera margaritifera* glochidia cause hyperplasia and fusion of the gill filaments (Treasurer and Turnbull 2000; Thomas et al. 2014) and lessen the mucous cells of the gills (Thomas et al. 2014), but it is not known whether these changes could increase trout’s resistance to *F. columnare*. Furthermore, the protective effect of *M. margaritifera* infection after the glochidia drop-off is especially surprising. Metamorphosed glochidia rupture the gill epithelium when detaching (Waller and Mitchel 1989), which should increase vulnerability to secondary infections — especially to bacteria. Exposure of fish to pathogens entering the host via gills increases with ventilation rate (e.g., Mikheev et al. 2014). Therefore, the observed protective effect of *M. margaritifera* glochidia against *F. columnare* could be contributed by behavioural changes induced by glochidiosis — especially the decreased locomotor activity of host fish Horký et al. 2014, 2019) — which would decrease the ventilation rate of fish.

In theory, the observed protective effect of glochidia may be an adaptive feature of *M. margaritifera* to increase its survival and fitness (Ziuganov 2005; Poulin 2010; Hughes et al. 2012; Gopko et al. 2015, 2017a). It is not unprecedented that a parasite would enhance the immune defence of its host (so-called apparent competition), or in some other way impair the ability of a second parasite/microbe to enter the host (e.g. Ashby and King 2017), but it remains unclear why the effect would last for several months after the glochidia are shed.

Whatever the mechanism, it is possible that any parasite, not just *M. margaritifera*, could produce the observed decreased pathogenicity/virulence. However, several studies have found a lowered resistance to bacterial infections in fish that were pre-infected with parasites (Kotob et al. 2016). Fish’s increased susceptibility to bacterial infection has been shown in co-infection by monogenean gill parasites (Busch et al. 2003; Zhang et al. 2015), tissue-penetrating trematode metacercaria (Pylkkö et al. 2006), fish lice (Bandilla et al. 2006; Lhorente et al. 2014) and different ciliated ectoparasites (Evans et al. 2007; Xu et al. 2009, 2012; Shoemaker et al. 2012). A chronic myxosporean parasite infection decreased the resistance of rainbow trout to bacterial disease even 12 months after exposure to the parasite (Densmore et al. 2004). Thus, *M. margaritifera* glochidia increasing the survival of brown trout during an *F. columnare* disease outbreak is a notable exception among co-infections between parasites and bacterial pathogens, urging further investigation into the relationship between fish and parasitic glochidia.

The present results are especially interesting when compared to other *F. columnare* co-infection experiments which included pre-infection with parasites. Both the experiment on rainbow trout infected with the crustacean skin parasite *Argulus coregoni* (Bandilla et al. 2006) and the one on goldfish pre-infected with the monogenean gill parasite *Dactylogyrus intermedius* (Zhang et al. 2015) resulted in an increased susceptibility to *F. columnare* infection. Both *A. coregoni* and *D. intermedius* are ectoparasites equipped with suckers or hooks that penetrate host epithelial cells, and they also feed on the host’s blood or epithelial cells. This can damage the skin or gill epithelium, potentially creating a gateway for secondary microbial invasions. In contrast, the 70-µm diameter glochidia of *M. margaritifera*, after their initial penetration into the fish’s gills, are surrounded by a cyst embedded within the gill tissue; they stay and develop there for up to 11 months before detaching (e.g. Salonen et al. 2017). Thus, the association of *M. margaritifera* infection with decreased pathogenicity/virulence of *F. columnare* seems to be exceptional. Hence, this finding would increase the interest and willingness of various stakeholders such as commercial fish farms, fishing authorities, fishery collectives and landowners to participate in the conservation activities involving infection of salmonids with *M. margaritifera*. In the light of the present results, the possible protective influence on the salmonid host by *M. margaritifera* against the very harmful *Flavobacterium* fish pathogen can be added to the list of potential beneficial services provided by freshwater mussels. Therefore, and because of the large *F. columnare* problems of fish farms, present results urge further studies with deepened focus, for example, on the immune parameters and gill histology to better understand the interaction between fish, bacterium and the glochidia.
